# Lymph node and pulmonary tuberculosis during upadacitinib treatment in a psoriatic arthritis patient

**DOI:** 10.1093/rap/rkac032

**Published:** 2022-05-13

**Authors:** Larissa Valor-Méndez, Bernhard Manger, Jochen Wacker, Arnd Kleyer, Georg Schett

**Affiliations:** 1 Department of Internal Medicine 3, Rheumatology and Immunology; 2 Deutsches Zentrum für Immuntherapie (DZI), FAU Erlangen-Nürnberg and Universitätsklinikum Erlangen, Erlangen, Germany

Key message

- Awareness of emergence of tuberculosis should also include patients treated with Janus kinase inhibitors.

**Fig. 1:**
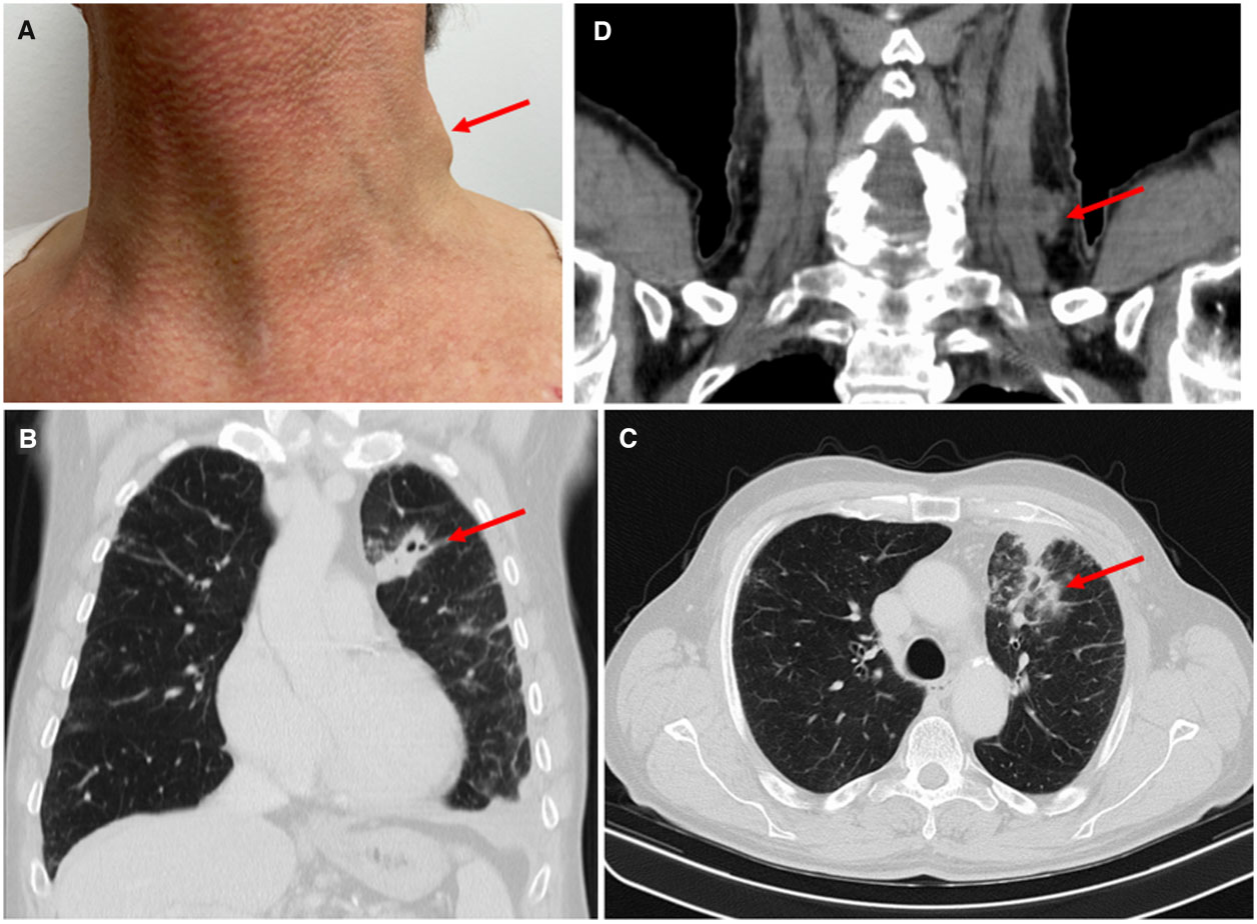
Lymph node and pulmonary tuberculosis during upadacitinib treatment (**A**) Tumour in the left side of the neck. (**B** and **C**) Consolidation in the left lung in CT scan. (**D**) Nodule in the left side of the neck in CT scan.

Dear Editor, We report on a 68-year-old Caucasian male with a 6-year history of PsA who developed pulmonary and lymph node tuberculosis (TB) 6 months after initiation of treatment with the Janus kinase 1 (JAK1) inhibitor upadacitinib. Before upadacitinib, he had been treated with MTX for 2 years, secukinumab for 10 months, ustekinumab for 6 months and infliximab for 12 months without success. The time interval between the last infliximab dose and the start of treatment with upadicitinib was 18 months. While being well controlled for his PsA with upadacitinib without concomitant synthetic DMARD, he presented with a rapidly and steadily growing tumour on the left side of his neck, which emerged within 2 weeks. Other symptoms, such as chills, fever, dyspnoea, cough, weight loss or night sweats, were absent. The patient was admitted to our ward for further work-up. On physical examination, he was haemodynamically stable and presented with a prominent lymph node on the left side of the neck. No other abnormalities were found. A chest radiograph and a chest/neck CT scan revealed a round consolidation in the left lung (Fig. 1B and C) and an enlarged lymph node in the left side of his neck (Fig. 1D). Laboratory data upon admission revealed normal white blood cell counts, mild anaemia (haemoglobin: 11.8–15.5 g/dl), elevated CRP (< 5 mg/l) and elevated ESR (< 20 mm/h). Serological tests for HIV, HBV and HBC were negative. IFN-γ release assay (IGRA) and nested PCR for *Mycobacteriumtuberculosis* complex in sputum were positive. Bronchoscopy, microscopy and culture of bronchoalveolar lavage fluid confirmed the presence of *M.* *tuberculosis*.

Our patient had no previous history of TB, close contact with anyone with TB, or travel to a TB-endemic area in the past decade. The screenings for latent TB using IGRA and chest radiographs were negative each time he was started on a new PsA treatment regimen. His last negative IGRA and normal chest radiograph were immediately before starting upadacitinib (Fig. 1A). After lymph node excisional biopsy that also confirmed TB, we started a quadruple therapy with ethambutol, rifampicin, isoniazid and pyrazinamide at standard dosages, because there was no evidence of genotypic resistance associated with the therapy. Owing to the known interaction via CYP3A4 between tuberculostatic drugs, upadacitinib was stopped. After 4 months of treatment, *M.* *tuberculosis* complex microscopy and culture in sputum were negative, and therapy was reduced to a combination of rifampicin and isoniazid. During TB treatment, the patient flared, with development of synovitis, morning stiffness, skin lesions and persistent nail psoriasis. We decided to re-administer upadacitinib after 5 months of TB treatment, because of prior good skin/joint disease control and the poor response to other biological DMARDs, under tight clinical and laboratory control, with rapid improvement of symptoms.

The diagnosis of TB remains challenging in the settings of older age, biological treatment and/or altered immune response. Anti-TNF antibodies are associated with an increased risk for reactivation of latent TB. Currently, no data regarding TB reactivation exist for upadacitinib [1]. Long-term safety data for tofacitinib (JAK1/3 inhibitor) [2, 3] and baricitinib (JAK1/2 inhibitor) [4] in RA revealed TB cases, mostly in endemic areas [5, 6]. To date, the safety profile of JAK inhibitors is characterized by an increased risk of herpes zoster infection, but not TB [5]. It has been hypothesized, based on other JAK inhibitors, such as ruxolitinib (JAK2), indicated for myelofibrosis and polycythaemia vera, that they might increase the risk of TB infections, through down-regulating Th-1 responses and production of IFN-γ, a key cytokine involved in protective immunity against *M.* *tuberculosis* [7].

In this case, TB started during upadacitinib treatment. It is unclear whether there is a causal relationship between TB and the treatment. However, there was no evidence for a new infection with TB, although it cannot be ruled out completely. Reactivation of TB seems more likely in this case, despite negative IGRA. False-negative IGRA tests have been described to occur during immune-modulatory treatment of arthritis [8]. This case highlights the limitations of screening for TB and the possibility that TB can complicate JAK inhibition. Tuberculostatic treatment, however, successfully contained the infection, also allowing the treatment with the JAK inhibitor upadacitinib to be restarted, showing that this complication could be managed well. Nonetheless, awareness of the emergence of TB should also include patients treated with JAK inhibitors.
